# Diagnostic value of urine sCD163 levels for sepsis and relevant acute kidney injury: a prospective study

**DOI:** 10.1186/1471-2369-13-123

**Published:** 2012-09-26

**Authors:** Longxiang Su, Lin Feng, Changting Liu, Zhaoxu Jiang, Ming Li, Kun Xiao, Peng Yan, Yanhong Jia, Dan Feng, Lixin Xie

**Affiliations:** 1 Department of Respiratory Medicine, Hainan Branch of the Chinese PLA General Hospital, Sanya, Hainan Province, 572013, China; 2 Medical College of Nankai University, Tianjin, 300071, China; 3 Department of Respiratory Medicine, Guangzhou Women and Children Medical Care Center, Guangzhou, 510623, China; 4 Department of Respiratory Diseases, Chinese PLA General Hospital, Beijing, 100853, China; 5 Department of Medical Statistics, Chinese PLA General Hospital, Beijing, 100853, China; 6 Nanlou Respiratory Diseases Department, Chinese PLA General Hospital, Beijing, 100853, China

**Keywords:** Urine, Soluble CD163 (sCD163), Sepsis, Systemic inflammatory response syndrome (SIRS), Prognosis, Acute kidney injury (AKI)

## Abstract

**Background:**

Sepsis is a common syndrome in critically ill patients and easily leads to the occurrence of acute kidney injury (AKI), with high mortality rates. This study aimed to investigate the diagnostic value of urine soluble CD163 (sCD163) for identification of sepsis, severity of sepsis, and for secondary AKI, and to assess the patients’ prognosis.

**Methods:**

We enrolled 20 cases with systemic inflammatory response syndrome (SIRS), 40 cases with sepsis (further divided into 17 sepsis cases and 23 severe sepsis cases) admitted to the intensive care unit (ICU), and 20 control cases. Results for urine sCD163 were recorded on the day of admission to the ICU, and AKI occurrence was noted.

**Results:**

On the day of ICU admission, the sepsis group exhibited higher levels of urine sCD163 (74.8 ng/ml; range: 47.9-148.3 ng/ml) compared with those in the SIRS group (31.9 ng/ml; 16.8-48.0, *P < 0.001*). The area under the curve (AUC) was 0.83 (95% confidence interval [CI]: 0.72-0.94, *P < 0.001*) the sensitivity was 0.83, and the specificity was 0.75 (based on a cut-off point of 43.0 ng/ml). Moreover, the severe sepsis group appeared to have a higher level of sCD163 compared with that in the sepsis group (76.2; 47.2-167.5 ng/ml vs. 74.2; 46.2-131.6 ng/ml), but this was not significant. For 15 patients with AKI, urine sCD163 levels at AKI diagnosis were significantly higher than those of the remaining 35 sepsis patients upon ICU admission (121.0; 74.6-299.1 ng/ml vs. 61.8; 42.8-128.3 ng/ml, *P = 0.049*). The AUC for urine sCD163 was 0.688 (95% CI: 0.51-0.87, *P = 0.049*). Sepsis patients with a poor prognosis showed a higher urine sCD163 level at ICU admission (98.6; 50.3-275.6 ng/ml vs. 68.0; 44.8-114.5 ng/ml), but this was not significant. Patients with AKI with a poor prognosis had higher sCD163 levels than those in patients with a better prognosis (205.9; 38.6-766.0 ng/ml vs. 80.9; 74.9-141.0 ng/ml), but this was not significant.

**Conclusions:**

This study shows, for the first time, the potential value of urine sCD163 levels for identifying sepsis and diagnosing AKI, as well as for assessment of patients’ prognosis.

**Trial Registration:**

ChiCTR-ONC-10000812

## Background

Sepsis is one of the major factors affecting the incidence of admission to the intensive care unit (ICU) and mortality in the ICU [1]. Sepsis, which rapidly progresses, is likely to be followed by secondary multi-organ dysfunction and endangers the patient’s life. The kidney is most susceptible to multiple organ failure. Therefore, the occurrence of acute kidney failure injury (AKI) lengthens hospital stay, decreases the inpatient’s budget, and increases mortality. From a clinical point of view, it is imperative to discover markers for early sepsis and AKI diagnosis, as well as for the patients’ prognosis, to determine an earlier intervention to save lives.

CD163 is a trans-membrane molecule, which has only been discovered on the membrane of mononuclear-phagocytes. CD163 is a specific scavenger receptor for hemoglobin/heme in the body, capable of specific recognition of the hemoglobin-haptoglobin complex. Studies in recent years have found that CD163 regulates the expression of anti-inflammatory molecules, such as interleukin-10 and heme oxygenase-1 [2,3]. Soluble CD163 (sCD163) comes from CD163 molecules, which peel off the membrane of mononuclear cells [2,4]. It has been reported that oxidative stress induced by H_2_O_2_ or a nitric oxide donor, as well as 8-iso-prostaglandin F2α, induce significant shedding of CD163 [5]. Feng et al [6] recently demonstrated that sCD163 is a sepsis diagnostic biomarker and can differentiate the severity of sepsis and the assessment of prognosis. Some studies have also reported high serum sCD163 expression in bacteremia patients and its value in prognosis [7,8], as well as high serum sCD163 expression in people with chronic kidney diseases [9]. CD163-Hb scavenger receptors play an important role in the process of clearance and conversion of hemoglobin/heme in chronic kidney disease [10]. Currently, it is still unknown whether sCD163 can be detected in urine and what value it possesses for sepsis and AKI.

Therefore, the present study investigated whether there are high sCD163 levels in urine with the occurrence of sepsis and AKI, as well as its clinical significance.

## Methods

All subjects were selected from inpatients hospitalized between May 2010 and September 2011 in the Respiratory Intensive Care Unit (RIUC), Surgical Intensive Care Unit (SIUC), and Emergency Intensive Care Unit (EICU) of the Chinese People’s Liberation Army (PLA) General Hospital. We obtained approval for the study from the Ethics Committee of the Chinese PLA General Hospital (project no. 20090923-001) and registered the study at the China Clinical Trials Register (ChiCTR-ONC-10000812). The subjects and their families were well informed of the details and signed relevant contracts prior to the study.

### Inclusion and exclusion criteria

In accordance with the 1991 ACCP/SCCM Sepsis Directory [11] and the diagnosis criteria advanced by the 2001 International Sepsis Definition Conference [12], systemic inflammatory response syndrome (SIRS) was diagnosed according to the following criteria: (1) temperature >38°C or <36°C; (2) pulse rate >90 beats/min; (3) ventilatory rate >20 breaths/min or hyperventilation with partial pressure of arterial carbon dioxide (PaCO2) <32mmHg; and (4) white blood cell count >12,000μL^-1^ or <4000μL^-1^, or >10% immature cells. Sepsis is a systemic inflammatory response to infection. Severe sepsis is defined as sepsis associated with organ dysfunction, hypoperfusion abnormalities, or sepsis-induced hypotension. When perfusion abnormalities cannot be corrected by adequate fluid resuscitation, septic shock occurs. In this study, we enrolled inpatients with two or more of the SIRS symptoms mentioned above within 24 h after ICU admission. Based on results from an etiological exam and three experienced doctors’ opinions, patients were divided into a SIRS group and a sepsis group. The sepsis group was also sub-divided into a sepsis group and a severe sepsis group (subjects with severe sepsis and septic shock), according to the severity of conditions. In addition, with 28-day survival as the cut-off, the patients were also grouped into survivors, subjects who stayed alive for 28 days (≥ 28 days), and non-survivors who died (< 28 days).

Exclusion criteria were as follows: (1) those under 18 years of age; (2) those who had suffered from anuria for a long time; (3) those with acquired immunodeficiency syndrome; (4) those who had reduced polymorphonuclear granulocytes (count <500μL^-1^); (5) those who were receiving dialysis treatment for chronic kidney disease; and (6) those who died within 24 h after being taken into the ICU, refused to become involved in the study, or gave up treatment during the period of observation.

### Renal function assessment and diagnosis of AKI

Plasma creatinine concentrations of patients were detected within 24 hours of being admitted to the ICU for baseline values [13,14]. Based on the 2006 Acute Kidney Injury Network (AKIN) definition, AKI involves different degrees of abnormal kidney structure and function, as well as signs of abnormal kidney damage lasting no more than 3 months, denoted clinically by blood, urine and tissue tests, and imaging studies. AKI includes acute renal failure, acute tubular necrosis, delayed graft function, is characterized by a 48-h increase in serum creatinine levels to ≥25.4 μmol/l (0.3 mg/dl), a percentage increase in serum creatinine levels of greater than 50%, or a 6-h (or longer) urine output below 0.5 ml·kg^-1^·h^-1^[15].

### Clinical data collection

Upon admission to the ICU, the following items were recorded for each patient: age, sex, chief complaints, symptoms, Acute Physiologic Assessment and Chronic Health Evaluation II (APACHE II) scores, Sequential Organ Failure Assessment (SOFA) scores, white blood cells counts (WBC), C-reactive protein (CRP), Procalcitonin (PCT) urine sCD163 levels, serum creatinine levels, blood urea nitrogen levels, urine output, mechanical ventilation, continuous hemodiafiltration, AKI, pathogens, underlying diseases, and time of admission. A record was also kept of the 28-day survival rate. Within 24 h after admission, intravenous blood was sampled, and urine was collected by a Folly catheter. Blood was centrifuged at 3000 rpm for 15 minutes, and the urine was centrifuged at 2000 rpm for 5 minutes. Supernatant (2 ml) from both was then transferred to a 1.5 mL micro-centrifugal tube and stored at -80°C.

### ELISA assays

Measurements of sCD163 concentration were obtained by double antibody sandwich ELISA (a soluble CD163 ELISA assay for the measurement of macrophage and monocyte activation, IQ Products, The Netherlands, product number: IPQ-383). Throughout the experiment, all the urine samples were diluted by 10 times before concentrations were measured. In this study, duplicate wells were used to detect urine sCD163 concentrations. Intra-assay variability was 3-6% and inter-assay variability was 5-8%. The minimum detectable concentration for the sCD163 assay was 0.23 ng/mL. Each step of examination was carried out in strict accordance with the product manual.

### Statistical analysis

Statistical analysis was performed with SPSS 16.0 (SPSS, Chicago, IL, USA). Quantitative data with normal distributions are denoted as mean ± standard deviation (SD). The Student’s t-test was performed to compare means between two groups. Quantitative data that were abnormally distributed were denoted as medians (25th and 75th percentiles) and the rank-sum test was conducted. Data of unordered categories were denoted by rate, and disparities between two groups were examined using a chi-square test. A receiver-operating characteristic (ROC) curve was employed to assess the diagnostic value of sCD163 levels in patients’ urine for sepsis, as well as sepsis-related AKI and its prognosis. The "optimal cut-off point" was the sCD163 concentration at the point on the ROC curve closest to (0, 1); i.e., to a 1-specificity of 0 and a sensitivity of 1. The related Pearson coefficient was used to prove the correlation between serum sCD163 and urine sCD163 concentrations.

## Results

### Characteristics of the patients

Eighty cases were included in the study, with 20 in the normal control group, 20 in the SIRS group, and 40 in the sepsis group. On the basis of 28-day survival, the sepsis patients were further divided into two sub-groups: survivors (20 cases) and non-survivors (20 cases). Table 1 shows the general condition of the subjects within 24 h after admission. We found that males were significantly more likely to contract sepsis than females (*P < 0.05*). Sepsis patients had significantly higher CRP and serum sCD163 levels, as well as APACHE II scores, than the SIRS patients (*P < 0.05*). Among the sepsis patients, the survivors had significantly higher serum sCD163 and blood urea nitrogen levels, as well as higher APACHE II and SOFA scores, compared with those of the non-survivors (*P < 0.05*). There were no differences in age, temperature upon admission, WBC, PCT, urine output, CCR, serum creatinine, ventilator use rate, pathogens, etiological examination results, and past medical history, either between the SIRS and the sepsis group, or between the survivors and the non-survivors.

**Table 1:** Clinical and biological data at admission in the intensive care unit

**Characteristics**	** SIRS**	**Sepsis**		
	** n = 20**	**Total n = 40**	**Survivors n = 20**	**Non-survivors n = 20**
**Age** (years)	55.4 ± 21.4	56.2 ± 19.8	52.7 ± 21.5	59.8 ± 17.8
**Gender** (n, %)				
Male	7 (35)	28 (70)	16 (80)	8 (40)
Female	13 (65)	12 (30)*	4 (20)	12 (60)
**temperature** (°C)	37.31 ± 0.6	37.70 ± 1.1	37.97 ± 0.9	37.43 ± 1.1
**WBC counts** (×10∧9/L)	11.7 (9.3-13.0)	10.5 (7.7-17.0)	10.1 (7.7-12.6)	12.6 (7.4-21.9)
**serum CRP** (mg/dl)	3.4 (1.7-6.0)	11.6 (4.3-17.1)*	8.4 (3.1-15.5)	13.1 (6.1-18.2)
**serum PCT** (ng/ml)	0.3 (0.2-1.4)	1.9 (0.4-7.4)	1.9 (0.2-7.9)	2.0 (0.6-7.9)
**serum sCD163** (ng/ml)	0.3 (0.2-0.4)	2.9 (1.6-11.3)*	1.7 (1.1-4.5)	10.2 (2.7-18.2)#
**Renal characteristics**				
Urinary output (ml/Kg/hr)	-	1.5 (1.1-1.9)	1.5 (1.0-1.9)	1.5 (1.1-2.1)
CCr(ml/min)	-	90.1 (38.5-120.3)	94.3 (65.7-141.5)	53.1 (31.2-107.0)
SCr(μmmol/L)	-	68.0 (55.8-130.7)	63.1 (53.6-96.6)	79.5 (62.1-110.1)
BUN(mmol/L)	-	9.2 (6.5-15.6)	7.7 (5.9-10.3)	11.5 (8.9-29.9)#
**APACHEII score**	11.8 ± 6.7	16.6 ± 7.3*	12.3 ± 6.4	20.9 ± 5.5#
**SOFA score**	-	7.9 ± 4.0	6.1 ± 2.9	9.6 ± 4.2#
**MV** (n, %)	13 (65)	31 (77.5)	14 (70)	17 (85)
**CRRT** (n, %)	1 (5)	12 (30)*	4 (20)	8 (40)
**Etiological factors** (n, %)				
Pulmonary infection	-	35 (87.5)	18 (90)	17 (85)
Abdominal infection	-	9 (22.5)	6 (30)	3 (15)
Urinary tract infection	-	11 (27.5)	8 (40)	3 (15)
Trauma/postoperative infection	-	16 (40)	10 (50)	6 (30)
Bacteremia	-	13 (32.5)	8 (40)	5 (25)
Catheter-related infections	-	8 (20)	5 (25)	3 (15)
Others	-	1 (2.5)	1 (5)	0 (0)
**Pathogens** (n, %)				
Gram-positive bacteria	-		11 (55)	8 (40)
Gram-negative bacteria	-		18 (90)	15 (75)
Fungi	-		11 (55)	11 (55)
**underlying diseases** (n, %)				
Hypertension	6 (30)	12 (30)	6 (30)	6 (30)
Diabetes	2 (10)	7 (17.5)	3 (15)	4 (20)
COPD	0 (0)	3 (7.5)	3 (15)	0 (0)
Coronary heart disease	3 (15)	4 (10)	2 (10)	2 (10)
Immunosuppressed	0 (0)	7 17.5)	2 (10)	5 (25)
Nervous system disease	0 (0)	3 (7.5)	2 (10)	1 (5)
CKD	1 (5)	7 (17.5)	3 (15)	2 (10)

Quantitative data of normal distribution are presented as mean ± SD. Quantitative data of non-normal distribution are presented as median (25th and 75th percentiles). Qualitative data are presented as n (%).

APACHE score, Acute Physiologic Assessment and Chronic Health Evaluation II scores; SOFA score, Sequential Organ Failure Assessment scores; MV, mechanical ventilation; CRRT, continuous renal replacement therapy; COPD, chronic obstructive pulmonary disease; CKD, chronic kidney disease.

### Urinary sCD163 concentrations for the diagnosis of sepsis

**Figure 1:**
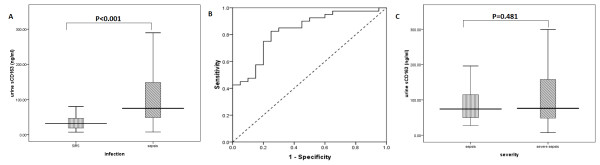
**Urine sCD163 levels on the day of admission to the ICU. A**, SIRS group compared with the sepsis group. **B**, Receiver-operating characteristic (ROC) curves for urine sCD63 levels for diagnosing sepsis. **C**, SIRS group compared with the severe sepsis group according to severity of sepsis. The boxes indicate the interquartile range (IQR). The dark lines denotes the median. The whiskers portray minimum and maximum values.

Urine sCD163 concentrations were not observed in the 20 people in the control group, meaning that they were below the limit of detection. First-day urine sCD163 concentrations were significantly higher in the SIRS group (20 cases) (74.8 ng/ml; range: 47.9-148.3 ng/ml) compared with those in the sepsis group (40 cases) (31.9; 16.8-48.0 ng/ml, *P < 0.001*) (Figure 1A). The area under the receiver-operating characteristic curve (AUC), which differentiated sepsis from SIRS, was 0.83 (95% confidence interval [CI]: 0.72-0.94, *P < 0.001*). With 43.0 ng/ml as the cut-off point, the sensitivity of urine sCD163 levels for the diagnosis of sepsis was 82.5% and specificity was 75% (Figure 1B). The sepsis patients were further divided into a sepsis group (17 cases) and a severe sepsis group (23 cases). Although the severe sepsis group appeared to have higher urine sCD163 levels than those in the sepsis group (76.2; 47.2-167.5 ng/ml vs. 74.2; 46.2-131.6 ng/ml), there was no significant difference between the two groups (*P = 0.481*, Figure 1C) .

### Urine sCD163 levels for the diagnosis of AKI

**Figure 2:**
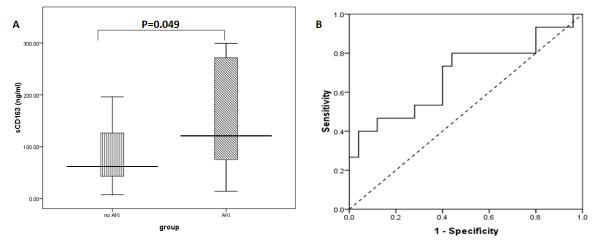
**Diagnostic value of urine sCD163 levels for acute kidney injury (AKI). A**, AKI group compared with the non-AKI group. **B**, Receiver-operating characteristic (ROC) curves for urine sCD63 levels for diagnosing AKI. The boxes indicate the interquartile range (IQR). The dark lines denote the median. The whiskers portray minimum and maximum values.

Fifteen out of the 40 sepsis patients developed AKI in the course of treatment. We compared urine sCD163 levels of these 15 patients with AKI with the remaining 35 patients upon ICU admission. Urine sCD163 levels in the AKI group were significantly higher compared with those in the non-AKI group (121.0; 74.6-299.1 ng/ml vs. 61.8; 42.8-128.3 ng/ml, *P = 0.049*) (Figure 2A). The AUC, which differentiated AKI from non-AKI, was 0.69 (95% CI: 0.51-0.87, *P = 0.049*). With 73.2 ng/ml as the cut-off point, the sensitivity of sCD163 levels for AKI diagnosis was 80% and specificity was 56% (Figure 2B).

### Urine sCD163 levels for prognostic assessment

**Figure 3:**
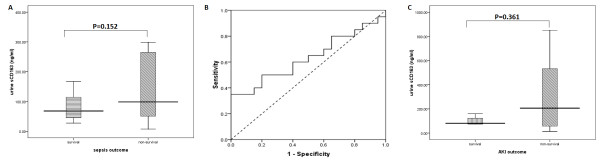
**The difference in urine sCD163 levels between survivors and non-survivors based on 28-day survival. A**, Patients with sepsis were divided into groups: the survivors group and non-survivors group. **B**, Receiver-operating characteristic (ROC) curves for urine sCD63 levels for the prognosis of sepsis. **C**, Patients with AKI were divided into groups: the survivors group and non-survivors group. The boxes indicate the interquartile range (IQR). The dark lines denote the median. The whiskers portray minimum and maximum values.

Upon admission, in the sepsis group, we found that urine sCD163 levels in the non-survivors were higher than those in the survivors [98.6; 50.3-275.6 ng/ml vs. 68.0; 44.8-114.5 ng/ml), but this difference was not significant (*P = 0.152*, Figure 3A). The AUC, which differentiated non-survivors from survivors, was 0.63 (95% CI: 0.46-0.81, *P = 0.152*) (Figure 3B). To more precisely determine the value of urine sCD163 levels for prognostic assessment, the AKI patients were further grouped into survivors (4 cases) and non-survivors (11 cases). Non-survivors appeared to have higher sCD163 levels than survivors (205.9; 38.6-766.0 ng/ml vs. 80.9; 74.9-141.0 ng/ml), but this was not significant (*P = 0.361*, Figure 3C).

### Correlation between serum sCD163 and urine sCD163 levels

**Figure 4:**
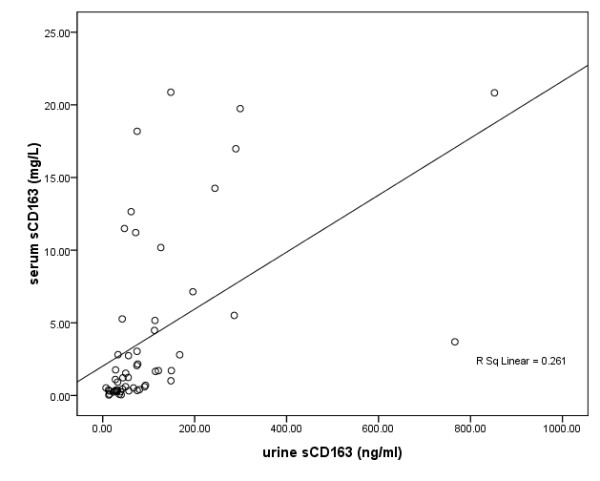
Correlation between urine sCD163 and serum sCD163 levels.

We correlated serum sCD163 and urine sCD163 levels from the 60 patients. A fairly good correlation was discovered between serum sCD163 and urine sCD163 levels, with a coefficient of 0.511 (*P < 0.001*, (Figure 4).

## Discussion

Sepsis is a common syndrome in critically ill people and is the leading precipitant of AKI [16], with mortality rates in excess of 70% [17,18]. Because of the importance of septic AKI, researchers have found many biomarkers for this syndrome, such as low-molecular-weight proteins (β2-microglobulin, α1-microglobulin, adenosine deaminase binding protein, retinol binding protein, cystatin C, and renal tubular epithelial antigen-1), enzymes (N-acetyl-β-glucosaminidase, alanine aminopeptidase, alkaline phosphatase; lactate dehydrogenase, α/π-glutathione-S-transferase, and γ-glutamyl transpeptidase), cytokines (platelet activating factor and IL-18), and other biomarkers (kidney injury molecule-1 and Na/H exchanger isoform-3) [19]. However, early diagnosis of AKI, especially septic AKI, is still a problem encountered by clinicians. Inflammation has an important role in the initiation of and the extension phases of AKI [20]. Therefore, biomarkers for inflammation and the mechanism of AKI have attracted much attention.

Hemoglobin/heme plays an important role in the occurrence of sepsis [6,21]. Pathological factors, such as hemolysis, disseminated intravascular coagulation, and ischemia reperfusion, as well as toxicity and side effects from drugs, result in release of hemoglobin/heme, yielding ferrous ions. At the same time, an oxidation reduction reaction catalyzing hydrogen peroxide leads to release of oxygen free radicals, causing damage to the kidney and other organs [22]. Therefore, it is necessary to clear hemoglobin/heme in a timely manner, preventing its excessive discharge into the blood and the outbreak of severe pathological responses. Haptoglobin takes part in the clearance of hemoglobin/heme and is involved anti-inflammatory metabolism, thus acting as a brake on relevant toxicity, and warding off oxidative damage. By means of quantitative urine mass spectrum analysis (unpublished data), we previously observed an increase of haptoglobin in the urine of patients with a poor prognosis. Vanhoutte et al [23] discovered a similar phenomenon while investigating AKI patients’ urine through a mass spectrum study (SELDI-TOF-MS). These findings suggest that hemoglobin/heme clearance occurs in parts of the kidney under pathological conditions. CD163 is the sole receptor mediating the clearance of hemoglobin/heme [24]. Hemoglobin/heme can only be cleared by phagocytes when combined with haptoglobin and through the medium of CD163 [25]. Soluble CD163 is a soluble form of CD163, and its expression is related to more than one disease [8,26-28]. Especially in recent years, reports have described a high expression of sCD163 in patients with chronic kidney diseases [9]. The scavenger receptor sCD163-Hb participates in the conversion and clearance of hemoglobin/heme in the course of chronic diseases [10]. For this reason, we speculate that urine sCD163 is likely to be highly expressed and reflects kidney injury and development of sepsis. However, there are no reports regarding this issue.

Despite the fact that a normal amount of hemoglobin/heme can be cleared locally in the kidney, our study failed to detect sCD163 in the urine from the control group, which suggests that the technique applied may not be sensitive enough to measure minute quantities of sCD163. However, this technique is still effective in the diagnosis of sepsis. In the current study, urine CD163 concentrations were abnormally increased during sepsis, with urine sCD163 concentrations in the sepsis group significantly higher than those in the SIRS group. Urine sCD163 concentrations also had an ideal sensitivity and specificity for the diagnosis of sepsis. With regard to assessment of severity of sepsis by urine sCD163 concentrations, despite the finding that urine sCD163 concentrations in the severe sepsis group appeared to be higher than those in the sepsis group, this difference was not significant. In addition, patients with a poor prognosis tended to exhibit higher urine sCD163 concentrations at an early stage, which is helpful for prognosis assessment. The non-survivors had higher urine sCD163 concentrations than the survivors. As sepsis progresses, delayed or ineffective treatment will finally lead to AKI. This explains the higher urine sCD163 concentrations in AKI patients compared with those in non-AKI patients. ROC efficiency analysis demonstrated good diagnostic value of urine sCD163 for AKI because AKI patients had significantly higher urine sCD163 concentrations than non-AKI patients. The AUC was 0.69, and the sensitivity and specificity were 80% and 56%, respectively. Based on the prognosis after 28 days, we grouped the AKI patients (15 cases in total) further, finding that after contracting AKI, the non-survivors had higher sCD163 levels than the survivors. Because high levels of urine sCD163 often suggest kidney injury and a bad prognosis, urine sCD163 could be employed to assess the prognosis in patients who develop AKI. In the present study, we found a moderate correlation between serum and urine sCD163 levels, rendering it possible to make a diagnosis of sepsis and monitor its development using urine sCD163 levels. In summary, the detection of sCD163 concentrations in sepsis and AKI patients leads us to hypothesize that urine sCD163 levels indicate renal function, they can be used to differentiate sepsis, and they are useful for the assessment of prognosis.

Sepsis may be complicated by renal dysfunction [29]. Ischemia and nephrotoxicity injury may interfere with hemoglobin/heme and protein complexes inside renal cells, leading to an increase of intercellular hemoglobin/heme [30,31]. In addition, hemoglobin/heme is produced by sepsis through the megalin/cubilin receptor [32]. It has been reported that saturation of albumin and/or damage of megalin/cubulin receptors appears to play a crucial role in the concentrations of several small molecules [33], which may result in higher hemoglobin/heme concentrations. Accumulation of hemoglobin/heme is nephrotoxic in two ways. First, hemoglobin/heme, through its pro-inflammatory role, mediates renal injury. With the enhancement of hemoglobin/heme levels in the body, adhesion molecules in the bowel, liver, kidney, and other organs are up-regulated, accompanied by leukocytic recruitment and greater vascular permeability [34-36]. Second, in this respect, cell toxicity exerts a direct effect. Plasma and membrane lipid composition are oxygenated by hemoglobin/heme, which causes protein denaturation, as well as damage to the integrity of the cytoskeleton. Hemoglobin/heme lowers the activity of some enzymes but stimulates the destruction of some cells. Hemoglobin/heme also leads to DNA oxidation and denaturation [37]. Localized hemoglobin/heme clearance proceeds inside the kidney. It is important to prevent irreversible damage to the kidney by this continuous process. Haptoglobin, combined with hemoglobin/heme, as well as with the scavenger receptor CD163 on the surface of phagocytes, and by means of endocytosis, stimulates the clearance of hemoglobin/heme. At the same time, CD163 molecules on the cell membrane peel off, forming a detectable sCD163. The current study found that when sepsis occurred or when it was complicated with AKI, sCD163 concentrations were higher than those in SIRS patients, whereas no sign of sCD163 was found in the urine of normal persons. Additionally, the CCR value of 15 patients with AKI was 42.3 ± 21.5 ml/min, which showed moderate damage to glomeruli. Glomerular injury is caused by localized inflammatory reactions in the kidney and oxidative stress, as well as by inducing epithelial and endothelial cell injury, and inducing changes in the glomerular pore diameter and charge barrier [20,38]. Soluble CD163 has a molecular mass of approximately 130,000 daltons, and an electronegative pI of 5.82 is found in the urine after a massive compromise of the glomerular barrier. This is also one of the possible mechanisms of increased sCD163 concentrations in the urine.

Our study is limited by the following. (1) Our sample size was limited. We found that although the severe sepsis group appeared to have higher sCD163 concentrations than the sepsis group and non-survivors also appeared to have higher sCD163 concentrations than survivors, this difference was not significant. The reason for this might be because of the limited sample size and the dialysis treatment that patients were exposed to. (2) The range of the study was limited. Our study focused on the clearance mechanism for hemoglobin/heme, with sepsis patients as subjects. Not just sepsis can induce AKI, with many other factors involved in causing AKI. It has been reported that septic AKI may be characterized by a distinct pathophysiology from non-septic AKI based on experimental and clinical data [39,40]. Therefore, it is important to determine whether the increase in biomarkers in AKI is independent of the increase due to sepsis [41]. However, we did not enroll patients with AKI without sepsis in this study. Further studies need to be conducted on hemoglobin-haptoglobin metabolism and sCD163 concentrations in the case of acute or chronic renal injury by other causes. (3) The study results may have been affected by dialysis treatment. Studies have shown that dialysis treatment gets rid of hemoglobin/heme. This suggests that sustained dialysis treatment, which some of the patients received, had an effect on their sCD163 concentrations. However, because of the fairly small sample size, we failed to exclude these patients from the study.

## Conclusions

This study suggests, for the first time, the potential value of urine sCD163 levels for identification of sepsis and diagnosis of AKI, as well as for early assessment of patients’ prognosis. As a noninvasive detection index, urine sCD163 levels may have potential clinical value, although this is yet to be proved by a larger clinical sample size.

## Abbreviations

ICU: Intensive care unit; AKI: Acute kidney injury; SIRS: Systemic inflammatory response syndrome; APACHE II score: Acute physiologic assessment and chronic health evaluation II score; SOFA score: Sequential organ failure assessment score; WBC: White blood cells; CRP: C-reactive protein; PCT: Procalcitonin; MV: Mechanical ventilation; CRRT: Continuous renal replacement therapy; COPD: Chronic obstructive pulmonary disease; CKD: Chronic kidney disease; ROC: Receiver-operating characteristic; AUC: Area under the curve; CI: Confidence interval.

## Competing interest

The authors declare that they have no potential conflicts of interest.

## Authors' contributions

LS and CL designed the study, acquired the data, managed the data analysis, and drafted the manuscript. LF substantially contributed to the interpretation of the data and critically revised the manuscript. CJ, ML and KX carried out the study in the SICU, EICU and the RICU, respectively. PY and YJ participated in the design of the study, and substantially contributed to the interpretation of the data. DF assisted in the data analysis and the use of medical statistics. LX was responsible for protocol revisions, data analysis, and final draft revision. All authors have read and approved the final manuscript.

## Pre-publication history

The pre-publication history for this paper can be accessed here:

http://www.biomedcentral.com/1471-2369/13/123/prepub
